# Two Cases of Traumatic Tetanus

**Published:** 1879-12

**Authors:** G. H. Salisbury


### Clinical Reports.

### NOTES FROM HOSPITAL PRACTICE.

### ARTICLE VIII.

*Two Cases of Traumatic Tetanus.*

*I.*—W. M., laborer; age 16; native of U. S.; was admitted to Cook County Hospital June 5th, 1879.

On admission, the patient gave the following history: His health had usually been good. In childhood, he had small-pox. One of his brothers is said to have died of tetanus.

Ten days previous to admission, he was kicked on the left side of the head by a horse. He did not lose consciousness, but, with the aid of a companion, walked to his home, a distance of 400 metres. Dr. E. W. Lee was called, who found an irregular, lacerated wound on the left side of the face. The wound was closed with interrupted needle sutures, and was dressed with a carbolic acid lotion. The needles were removed the third day. The wound did well, and everything was favorable, until June 1st, six days after the injury, when Dr. Lee saw him, and found no unfavorable symptoms. On the 31st day of May, it may be noted, there was a marked fall in the atmospheric temperature. To this may possibly be attributed some agency in bringing on the tetanus. Soon after Dr. Lee saw him on the 1st of June, the symptoms of tetanus began, with slight stiffness about the jaw, and slowly progressed until June 4th, when the patient came to Dr. Lee's office. He had then had some difficulty in swallowing for a day or two. Dr. Lee recognized symptoms of tetanus, and next day brought him to the hospital. When admitted, the patient was fairly nourished. He complained of stiffness of the muscles of the neck. He answered slowly and with difficulty to questions. When he attempted to speak, the muscles of the mouth were thrown into spasmodic action, the features assuming at times a true risus sardonicus. He could only separate his teeth about one centimeter, and swallowed with difficulty, in consequence of the spasmodic action of the muscles of deglutition. The circulation was rather feeble and the extremities were cold and bluish. The wound was in a healthy condition.

The patient was placed in bed and rendered comfortable. His temperature on admission was 36.6; pulse 84 per minute, and somewhat feeble.

A cathartic was at once given. He also received:

| | | | |
|---|---|---|---|
| ℞ | Potassi bromidi | 3 | 00 |
| | Chloral hydrates | 1 | 00 |

every four hours. Good nourishment was given, in the form of milk, beef tea, etc. A record of the temperature was kept, which was:

| | |
|---|---|
| June 6th | P. 80, T. 36.3 |
| 6th, p. m | P. 76, T. 37 |
| 7th | P. 72, T. 36.5 |
| 7th, p. m | P. 72, T. 37 |
| 8th, p. m | P. 76, T. 37.1 |
| 9th | P. 88, T. 37 |

The stiffness of the muscles was constant, and slight spasms, affecting chiefly the muscles about the mouth and those of the neck, were excited upon attempting to speak or to swallow.

June 11th, he had an attack in which there occurred spasmodic closure of the mouth and spasm of the respiratory muscles, so that he became quite blue in the face. At the same time, there was bending back of the head and spasmodic action of the muscles of the back and extremities, producing slight opisthotonos. After the nurse pried open his mouth with a spoon, he breathed easier, and the spasm passed away. At noon of June 11th, he had another attack, exactly similar to the one just described. He passed the remainder of the day and night and the day of June 12th without any more serious symptoms than he had before the 11th. His temperature was normal, but his pulse was somewhat quickened. In the evening of the 12th, he had an attack more severe than any of the preceding. After this attack, 8 grams of bromide of potassium were at once given, followed by 2 gram doses every two hours. He was quiet during the night and slept some. On the next morning, he had a fourth attack.

As the case threatened to become very serious, the hypodermic injection of calabar bean was begun. The following solution was used:

| | | |
|---|---|---|
| ℞ Ext. Physostigmatis | | 50 |
| Glycerinæ | 8 | 00 |
| Aquæ ad | 32 | 00 |

M. Sig.—Inject 1 C. C. every two hours.

1 C. C. of this solution contains about 0.015 grams of the extract. The use of the bromide and chloral was also continued.

From this time, he improved considerably. No more paroxysms occurred. Although the calabar bean was used until there was considerable depression of the circulation, no contraction of the pupils occurred.

The wound healed rapidly, and he was discharged recovered on the 30th of June. His pulse was somewhat rapid after the violent attacks began, ranging from 96–124 per minute, but his temperature remained about normal.

*II.*—Of the second case I have not full notes, but the history, omitting dates, is briefly as follows. The patient, about a week before admission, received a crushing injury of the foot without any laceration of the skin.

About 36 hours before admission, he began to show signs of tetanus, and received a mixture of bromide of potassium and hydrate chloral.

After admission to the hospital, he received hourly 0.008 grams of the alcoholic extract of calabar bean by hypodermic injection, and 16 grams of whisky by the mouth, and once in two hours he received bromide of potassium 2 grams, and hydrate of chloral 1 gram. In addition, he had plenty of beef-tea and other nutriment. The paroxysms which amounted to slight opisthotonos, occurred once in half an hour, and at other times, in consequence of irritation. About 48 hours after admission, and eight hours before death, he began to have difficulty in swallowing, and some hebetude of mind. About an hour before death he had a somewhat severe paroxysm, followed by rapid breathing, rapid and feeble pulse, and unconsciousness. Tracheal rales were soon heard, and death occurred in about an hour. No more marked paroxysms occurred, but there was still some tonic spasm of the muscles shortly before death.

A most interesting question is suggested to me by these two cases, viz., what shall we take as evidence of the toxic effect of calabar bean poisoning in cases of tetanus?

In the first case, while symptoms of marked depression occurred, no contraction of the pupil was obtained. Was the depression due to the calabar bean?

In the second case, the manner of death was very curious. The spasms had not been severe and long continued enough to produce fatal exhaustion, and there were no signs of any considerable asphyxia. Could the cause of death in this case be calabar bean poisoning? If so, why did not relaxation of the muscles occur?

It is conceivable that in tetanus the activity of the spinal cord may be so increased, that an amount of calabar bean sufficient to paralyze the heart, may be insufficient to produce relaxation of the spasms.

G. H. SALISBURY.

---

TRI-STATE MEDICAL SOCIETY.—At the recent meeting of this Society, composed of members from Illinois, Indiana and Kentucky, Dr. H. B. Buck, of Springfield, was chosen president, and Louisville, Ky., selected as the place for holding the next annual meeting. The recent meeting at Evansville was well attended, and many interesting papers were read. The paper that will probably attract more attention than any other was read by Dr. Reuben A. Vance, of Cincinnati, on lithotrity.

SPECIALTIES of practice are one thing, and in the main, we think, undesirable; specialty in study is quite another matter.—*London Lancet.*

